# Topological Analysis of γH2AX and MRE11 Clusters Detected by Localization Microscopy during X-ray-Induced DNA Double-Strand Break Repair

**DOI:** 10.3390/cancers13215561

**Published:** 2021-11-05

**Authors:** Hannes Hahn, Charlotte Neitzel, Olga Kopečná, Dieter W. Heermann, Martin Falk, Michael Hausmann

**Affiliations:** 1 Kirchhoff Institute for Physics, Heidelberg University, 69120 Heidelberg, Germany; hannes_hahn@web.de (H.H.); charlotte.neitzel@osnanet.de (C.N.); 2 Institute of Biophysics of the Czech Academy of Sciences, 612 65 Brno, Czech Republic; olga.kop@centrum.cz (O.K.); falk@ibp.cz (M.F.); 3 Institute for Theoretical Physics, Heidelberg University, 69120 Heidelberg, Germany; heermann@tphys.uni-heidelberg.de

**Keywords:** DNA double-strand breaks, topology of γH2AX clusters, topology of MRE11 clusters, persistent homology, single-molecule localization microscopy, ionizing photon irradiation

## Abstract

**Simple Summary:**

Super-resolution single-molecule localization microscopy (SMLM) allows the detection of cluster formation of phosphorylation sites and recruited repair proteins (e.g., MRE11) around DNA double-strand breaks induced by ionizing radiation. The spatial arrangement of these targets can be described by distance frequency distributions, which can be used as a measure for cluster size and the ratio of cluster numbers per specimen analyzed. For the analysis of the shape of clusters and their topology and similarity, persistent homology was applied and the features were compared along the DNA repair process for two different cell lines (MCF-7 breast cancer cells and CCD-1059SK human skin fibroblasts). The similarity values for components and holes were averaged for each repair time point post-irradiation so that it was possible to compare not only clusters with each other but also cell specimens of different repair periods with each other. It was shown that after X-ray irradiation, these similarity values were high for γH2AX clusters in the early repair phase, while MRE11 clusters revealed an increased similarity at later time points.

**Abstract:**

DNA double-strand breaks (DSBs), known as the most severe damage in chromatin, were induced in breast cancer cells and normal skin fibroblasts by 2 Gy ionizing photon radiation. In response to DSB induction, phosphorylation of the histone variant H2AX to γH2AX was observed in the form of foci visualized by specific antibodies. By means of super-resolution single-molecule localization microscopy (SMLM), it has been recently shown in a first article about these data that these foci can be separated into clusters of about the same size (diameter ~400 nm). The number of clusters increased with the dose applied and decreased with the repair time. It has also been shown that during the repair period, antibody-labeled MRE11 clusters of about half of the γH2AX cluster diameter were formed inside several γH2AX clusters. MRE11 is part of the MRE11–RAD50–NBS1 (MRN) complex, which is known as a DNA strand resection and broken-end bridging component in homologous recombination repair (HRR) and alternative non-homologous end joining (a-NHEJ). This article is a follow-up of the former ones applying novel procedures of mathematics (topology) and similarity measurements on the data set: to obtain a measure for cluster shape and shape similarities, topological quantifications employing persistent homology were calculated and compared. In addition, based on our findings that γH2AX clusters associated with heterochromatin show a high degree of similarity independently of dose and repair time, these earlier published topological analyses and similarity calculations comparing repair foci within individual cells were extended by topological data averaging (2nd-generation heatmaps) over all cells analyzed at a given repair time point; thereby, the two dimensions (0 and 1) expressed by components and holes were studied separately. Finally, these mean value heatmaps were averaged, in addition. For γH2AX clusters, in both normal fibroblast and MCF-7 cancer cell lines, an increased similarity was found at early time points (up to 60 min) after irradiation for both components and holes of clusters. In contrast, for MRE11, the peak in similarity was found at later time points (2 h up to 48 h) after irradiation. In general, the normal fibroblasts showed quicker phosphorylation of H2AX and recruitment of MRE11 to γH2AX clusters compared to breast cancer cells and a shorter time interval of increased similarity for γH2AX clusters. γH2AX foci and randomly distributed MRE11 molecules naturally occurring in non-irradiated control cells did not show any significant topological similarity.

## 1. Introduction

DNA damage induced by ionizing radiation and the follow-up repair processes of damage sites depend not only on the radiation type, the applied dose, the dose rate, and the linear energy transfer (LET) but also on the cell radio-sensitivity, its DNA repair capacity, etc. (for review, see [1,2]). In contrast to high-LET particles (e.g., α-particles, heavy ions, etc.) passing and damaging the chromatin in linear tracks [3,4], low-LET photon irradiation induces DNA lesions in a coarse-grain pattern, which locally leads to single-strand breaks (SSBs) and double-strand breaks (DSBs) randomly distributed in the cell nucleus [2,5,6,7]. 

The most severe damages being created are double-strand breaks (DSBs) of the DNA molecule, which can be caused by several molecular-breaking mechanisms at a damage site [8]. In general, one of the first reactions by which the genome responds to DSBs is the specific phosphorylation of histone H2AX at ser139 in a chromatin region of 2 mega base-pair (Mbp) DNA surrounding the damage site [9]. Using specific antibodies against these phosphorylation sites, DSBs can be visualized in the form of so-called γH2AX foci [10,11]. For low-LET irradiation, the number of γH2AX foci is proportional to the number of DSBs [11], which can be assumed to follow a linear or linear-quadratic dependency to the dose applied [12]. Thus, microscopic counting of specifically labeled γH2AX foci has become a procedure well established in biological dosimetry nowadays [13] with a high degree of standardization and automation [14].

Novel techniques of super-resolution fluorescence microscopy, such as stimulated emission depletion microscopy (STED) or single-molecule localization microscopy (SMLM) [15,16], have been applied to repair focus analysis mostly in fixed specimens (see, e.g., [1,2]) but also in living cells [7,17]. Recent publications have shown that visible micrometer-sized γH2AX foci reveal a regular sub-structure [12,18,19,20], which is typically organized into three to four clusters. Our recent investigations carried out for different cell lines, e.g., HeLa cells, breast cancer cells, and fibroblasts, pointed out that these γH2AX clusters are about equal in size (typically about 400–500 nm in diameter) as long as they persist after DNA damage induction [19,21]. The number of clusters as well as the number of labeling points detected by SMLM follow a linear quadratic dose effect dependency, too [12]. γH2AX clusters were not only found to be similar in size but also showed a characteristic feature in shape [22,23]. When we studied the point topology of the clusters by scale-invariant persistence homology calculations, we found that the clusters closely associated with heterochromatin regions show a high degree of topological similarity [24,25]. Together with the findings that DSBs induced in densely packed heterochromatin are exposed to the border of the heterochromatic region in order to be repaired [26,27,28,29], our findings concerning the size and shape of γH2AX clusters indicate that the outcome of H2AX phosphorylation is not random and the nano-architecture of the γH2AX clusters should have an impact on the repair mechanism to be initiated at the given damage site.

The main DSB repair pathways following γH2AX cluster formation are canonic non-homologous end joining (c-NHEJ), homologous recombination (HR), and less precisely classified alternative (or backup) pathways, such as Ku-independent non-homologous end joining (a-NHEJ), single-strand annealing (SSA), and microhomology-mediated end joining (MMEJ) [1,2,30,31,32,33,34,35,36]. All these repair pathways follow different principles, need different times for DNA strand reconstruction, and recruit different series of repair proteins to the initially induced γH2AX clusters. Beyond the repair speed, a main difference in the pathways is the quality of repair. HR is precise in reconstructing the original base sequence of the damaged region. The process, however, is slow, since a complementary DNA sequence template has to be arranged, the damaged strands have to be resected, and the resected sequence has to be reconstructed again nucleotide by nucleotide. In contrast to HR, c-NHEJ is much faster and tolerates the loss of some nucleotides in the re-joint strand. After trimming of the broken ends, they are just fixed together. a-NHEJ and other alternative pathways are usually slow and error prone. They only seem to become relevant after multiple damages by high-dose exposures, when broken DNA ends are repaired differently due to a lack of c-NHEJ and HR repair proteins.

The decision of a cell about which repair pathway to use at a given damage site appears to be multi-factorial. The repair pathway selection seems to depend on several conditions, such as the cell cycle phase, the local chromatin structure and compaction, the genetic function at the site of damage, the complexity and multiplicity of DSBs in the whole nucleus, the general radiation sensitivity of the cell type, and other factors [30,37,38,39,40,41,42,43,44]. All these factors contributing to repair pathway choice at a given damage site have to be immediately considered by a cell. The mechanism responsible for integration and consideration of all these factors is not known yet but might be based on a complex epigenetic controlling system [5,6], which may interact according to rules not completely understood so far. Beyond the classical epigenetic protein pathways, another epigenetic control mechanism may therefore become relevant. Such an epigenetic system, not pushed into the focus of research yet, may be the architecture of chromatin networks and consequently the architecture of damage sites or γH2AX clusters. In general, the chromatin architecture is known to be functionally organized [45,46] on the micro-, meso-, and nano-scale, and its relaxation and re-arrangements were observed at damage sites [28]. Understanding how geometric arrangements of chromatin and topological organization of recruited proteins impact DNA repair processes requires a precise analysis of geometry and topology, not only of single molecules of γH2AX, but also of proteins forming repair complexes (foci and clusters) at DSBs, e.g., MRE11, 53BP1, and RAD51. 

A prominent DNA strand resection and broken-end-bridging component that is involved in HR repair and, especially at high doses, in a-NHEJ is the MRE11–Rad50–Nbs1 complex, known as the MRN complex (reviewed in [47]). MRN binds both nucleosomal homoduplex DNA and free DNA ends, activates ATM [48,49], and recruits EXO1 for end processing [50]. MRE11 is part of the MRN complex and has been one of the first DNA repair proteins detected at DSBs in vivo [51]. MRE11 is known for being associated with DSB ends in the amount of only a few proteins [50]. It might be assumed that these molecule clusters mark DSB ends containing MRN (MRE11) molecules engaged in DSB processing.

In our earlier study, where cells were exposed to high-LET radiation [52], MRE11 formed clusters embedded within larger clusters of γH2AX. The diameter of MRE11 clusters was about the half of that of γH2AX clusters. Interestingly, a similar diameter of MRE11 clusters (up to about 200–250 nm) was also observed in MCF-7 breast cancer cells and CCD-1059SK human skin fibroblasts exposed to low-LET 6 MeV X-rays (2 Gy), as shown in a previous article of this data set [21]. The results of this article have been the basis for the investigations presented here. The performance of the data set originally acquired in [21] motivated us to develop and apply novel cluster and topology algorithms [24] in order to investigate the similarity of γH2AX and MRE11 clusters and their dynamic changes during a long period of time (48 h) after irradiation.

## 2. Materials and Methods

Since the present article is a follow-up extension of our previous article [21], also published in the *Cancers* journal, Section 2.1 only briefly recapitulates the Materials and Methods section of the original article in order to avoid self-plagiarism. For details on the cell preparation, irradiation, SMLM instrumentation, and data acquisition, the reader is referred to [21]. The novel methods developed and applied in this article are described in detail in Section 2.2, Section 2.3, Section 2.4, Section 2.5 and Section 2.6.

### 2.1. Cell Preparation, Irradiation, and SMLM Acquisition of the Data Set

Cells of the human breast cancer cell line MCF-7 established from a pleural effusion of a 69-year-old female having an aneuploidy karyotype with stable MRE11 overexpression and cells of the human skin fibroblast cell line CCD-1059SK established from a biopsy of a 20-year-old female were cultivated, as described in detail in [21]. The cells were irradiated with the linear accelerator Artriste (Siemens, Erlangen, Germany) using 6 MV photon energy at a radiation dose of 2 Gy and a dose rate 3 Gy/min. The irradiated cells were further cultivated, and aliquots were fixed at defined time points (10 min, 30 min, 60 min, 120 min, 180 min, 24 h, and 48 h) post-irradiation. Then the cells were labeled by a primary mouse anti-γH2AX antibody (Merck, Darmstadt, Germany) with a secondary AlexaFluor 568 goat anti-mouse antibody (Invitrogen, Thermo Fisher Scientific, Schwerte, Germany) and a primary rabbit anti-MRE11 antibody (Abcam, Berlin, Germany) with a secondary AlexaFluor 488 goat anti-rabbit antibody (Invitrogen, Thermo Fisher Scientific, Schwerte, Germany). Details of the labeling protocol are described in [21]. For SMLM, the specimens were embedded in ProLong^®^ Gold (Thermo Fisher Scientific, Schwerte, Germany) antifade embedding medium, sealed, and stored for 24 h at 4 °C until ProLong^®^ Gold was polymerized.

SMLM was performed using a localization microscope with high thermomechanical stability, which is described elsewhere [12,19,21,53]. For each cell nucleus, a time stack of 2000 image frames was acquired and saved. From these images, local positions of the detected dye molecules were determined (see [19]) and the signal amplitude, the lateral x- and y-coordinates, the standard deviations in x- and y-directions, position errors, etc., were stored in a so-called orte-matrix [19,53], which was used as a basis for the data processing applied here.

### 2.2. Cluster Evaluation

For the analysis described here, the cluster determination program used in [21] could not be applied, because it does not provide a list of the coordinates of the detected points in clusters, which is necessary for topological evaluation.

Therefore, cluster formation had to be verified using the Density-Based Spatial Clustering of Applications with Noise (DBSCAN) algorithm [19], a widely used algorithm for cluster detection, which slightly differs from the algorithms applied in [21]. DBSCAN takes a set of points and uses two input parameters to divide all points into three categories: core points, density-reachable points, and noise points. With the two parameters ε and N_min_, point A is identified as a core point if there are at least N_min_ points in the circle around A with radius ε. Every point that is not a core point but whose distance to the nearest core point is ε or less is considered a density-reachable point. Every point that is neither a core point nor a density-reachable point is considered a noise point. Every core point and every density-reachable point is part of a cluster. As there is normally more than one cluster per set of points, the algorithm has to identify multiple clusters at once. To achieve this, two core points with a maximum distance of ε from each other are assigned to the same cluster, together with their respective density-reachable points. In this way, different clusters can be detected in the same set if they do not have core points with a distance of ε or less from each other or core points that share a density-reachable point.

**Figure 1:**
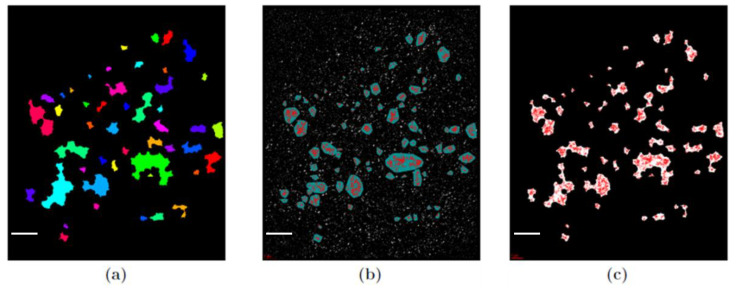
Example of images of γH2AX clusters after 180 min after 2 Gy irradiation of human skin fibroblasts. (**a**) Clusters detected by the original program used in [21]. (**b**) Cluster image obtained with the DBSCAN algorithm (cluster parameter values: N_min_ = 110; ε = 200 nm). (**c**) The same image as (**b**) but with the closing function applied. The high co-incidence of (**a**) and (**c**) can be observed. Scale bar: 2 µm.

With the in-house software 1-channel-analysis (Gote, Neitzel et al., manuscript in preparation), DBSCAN was applied on the data set. Various types of information for each cluster were obtained, such as the number of points detected per cluster, the cluster area, and the cluster perimeter, as well as information about the whole cell, such as the number of clusters detected per cell. To obtain reliable cluster detection, appropriate values for ε and N_min_ were interactively determined and compared to the cluster analysis used in [21]. In addition, 1-channel-analysis has the option to blind out the background by applying a closing function on the original image. Using this option, cluster analysis could be verified. In Figure 1, a representative example is presented. This shows clearly that the same points are identified as cluster points for the two programs. 

In the following topological analyses, the parameter values N_min_ = 110 and ε = 200 nm were used for γH2AX cluster determination and N_min_ = 60 and ε = 100 nm for MRE11 cluster determination. These values were not varied for the two cell lines. The next step was to create a list for each sample in which every cluster, together with the coordinates of all its signals, was included. 

### 2.3. Persistence Homology, Barcode Calculation, and Topological Similarities

To express the topological similarity of two clusters, it is important to not just compute the spatial overlap. This would compare in some way the geometry of the clusters but not the structure. With persistent homology [22,23], it is possible to simultaneously compare two clusters on a topological and geometrical level as both the internal structure and geometrical properties such as size and shape, matter in the context of biology. The first application of this method to γH2AX foci is well described in [24,25].

A central feature of this approach is the calculation of a barcode pattern as a representation of the pointillist form of clusters. Briefly, consider a set of n points. Put a circle with increasing radius, starting at zero, around each point. As soon as the circles of two points p and q intersect, draw a line from p to q. This object is considered as a component. If further circles intersect, a closed area can be formed, a hole. With increasing radius, increasingly more lines are closed and triangles closing a hole are formed until all points are connected with each other.

One can plot the number of components as a function of the radius. At the beginning or at radius zero, there are n components as there are n individual points. As soon as two individual components combine, as a result of increasing radius, they merge and form one single component, represented by the line connecting the two points. This represents the “death” of one component, resulting in n−1 components left. In a barcode the “birth”, in this case the starting point at zero, and the “death” of each component are depicted. With increasing radius, regions surrounded by lines but not filled with triangles are formed. These are called holes and can, just like components, be plotted as a function of the radius into a barcode. The “birth” of a hole is as soon as a polygon with more corners than a triangle forms, and it “dies” if it is filled with triangles. The holes start forming in the process of increasing radius, so their barcodes all start somewhere in the middle and end at the latest in the last bar of the components. The barcodes for the components are considered as barcodes of dimension zero and for the holes as barcodes of dimension one. With this method, a barcode characterizes the cluster on every size scale.

### 2.4. Topological Similarities

To determine the similarity of two clusters, their respective barcodes were compared to each other. A measure of the similarity of any two surfaces *M* and *N* can be computed by the Jaccard index [54]:

(1) $$J\left(M,N\right)=\frac{\left|M\;\cap^{\;}\;N\right|}{\left|M\;\cup^{\;}\;N\right|}$$

The Jaccard index can be used to compute a formula to measure the similarity *S* of two barcodes *A* and *B* consisting of bars a and b, respectively. This was shown in [24] and resulted in the formula given below:

(2) S(A,B)=1|A|+|B|[ ∑a∈A bϵBsup|a ∩  b||a ∪  b|+∑b∈B aϵAsup|a ∩  b||a ∪  b|]

The first summand in the brackets takes for a bar a in *A* the bar *b* in *B* for which the Jaccard index *J*(*a*,*b*) is the highest one. This is expressed by the supremum  b∈Bsup of the fraction $\frac{\left|a\;\cap^{\;}\;b\right|}{\left|a\;\cup^{\;}\;b\right|}\;$. This is done for all bars *a* in *A*, and the results are then summed up. Analogously, this is repeated for barcode *B* in the second summand. To normalize the equation, both sums are multiplied with the factor $\frac{1}{\left|A\right|+\left|B\right|}$. As a consequence, *S* has a highest possible value of 1, which represents two identical barcodes. The minimal value is 0, representing no overlap at all and as a consequence no similarity between the two barcodes and clusters.

Applying the described steps, a representation of the data coming from the orte-matrix is calculated in a way that each individual cluster barcode can be compared with another for the control group and the irradiated group of every cell line and for every repair period (10 min, 30 min, 60 min, 120 min, 180 min, 24 h, and 48 h) separately.

### 2.5. Heatmaps

To visualize the values of similarity between all clusters for each sample in an illustrative way, a so-called heatmap representation is computed for every two pairs of samples. A heatmap is a two-dimensional diagram with the cluster numbering of the measurements as an abscissa or ordinate. The numbering of the clusters is chosen in a way that clusters of the same nucleus are located next to each other. To display the similarity of two clusters, a color bar can be designed, assigning a specific part of the similarity interval [0;1] to a color spectrum. The similarity of two clusters *A* and *B* is then represented by the color of the point (*A*,*B*). In this way, a heatmap (1st-generation heatmap) for the components (dimension zero), the holes (dimension one), and the average of both is computed for every two pairs for samples. This results in many individual heatmaps, which in some cases include a few thousand clusters. 

First-generation heatmaps are becoming too complex and extended to obtain an overview and comparison of larger samples. Thus, a compact representation of the cluster similarities of different samples was computed by calculating the mean heatmap for each sample and creating a new heatmap composed of these averaged heatmaps (2nd-generation heatmap). Using such new heatmaps with the individual repair times (10 min–48 h) as the abscissa and ordinate and a color bar assigned to every pair of repair times, a dynamics in the formation and relaxation of similar clusters can be visualized.

From the perspective of biology, the averaged heatmaps may be seen as the relevant ones. In the case of the 2nd-generation heatmaps, which are the result of two averaging processes, the averaging of the components’ heatmaps and the averaging of the holes heatmaps, we show both results and in addition their average 2nd-generation heatmap.

### 2.6. Confocal Microscopy

For a visual impression of the specimens, confocal microscopy images of a few cells after immunofluorescence staining were captured using an automated Leica DM RXA microscope (Leica, Wetzlar, Germany). This instrument was equipped with a Plan Fluotar oilimmersion objective (100×/NA1.3), a CSU 10a Nipkow disc (Yokogawa, Japan), a CoolSnap HQ CCD-camera (Photometrix, Tuscon, AZ, USA) for detection, and an Ar/Kr-laser (Innova 70C Spectrum, Coherent, Santa Clara, CA, USA) for illumination.

## 3. Results

### 3.1. Cluster Formation

Cells of the human breast cancer cell line MCF-7 and the human skin fibroblast cell line CCD-1059SK were irradiated with 6 MeV photons at a dose of 2 Gy. At given times (10 min, 30 min, 60 min, 120 min, 180 min, 24 h, and 48 h) after irradiation, aliquots of the samples were taken, γH2AX and MRE11 were antibody-labeled, and the specimens were subjected to SMLM [21] followed by cluster analysis. As a control, the same cultures without irradiation were used and aliquots were subjected to the same procedures at the same time points.

**Figure 2:**
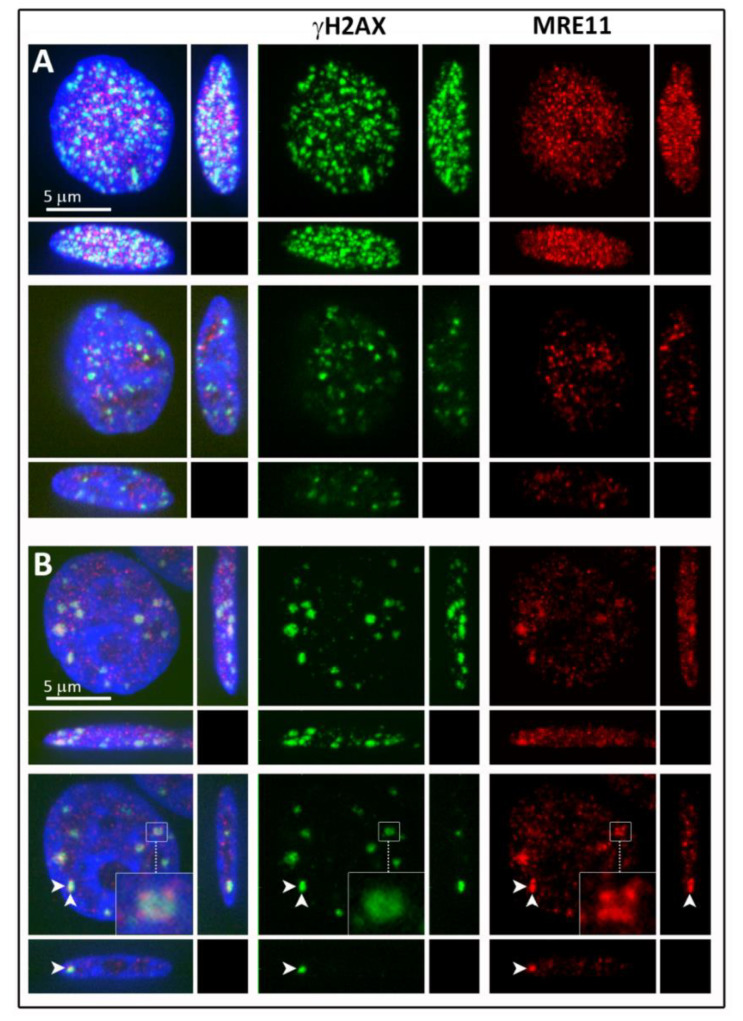
Confocal images of γH2AX (green) and MRE11 (red) focus formation in breast cancer cells exposed to 2 Gy of γ-radiation and left repair DSBs for 30 min (**A**) or 4 h (**B**). The top line of both panels represents the maximum immunofluorescence confocal microscopy images composed of 40 optical confocal slices (0.2 µm thick) and shown in all three x-y, x-z, and y-z planes. The bottom lines show a single confocal slice through the nuclei displayed in the top lines. For the post-irradiation period of 4 h, the inserts show a selected γH2AX+MRE11 focus in a magnified view. The white arrows indicate the position of x-y and y-z confocal planes. Chromatin counterstaining was performed with DAPI (artificially blue).

**Figure 3:**
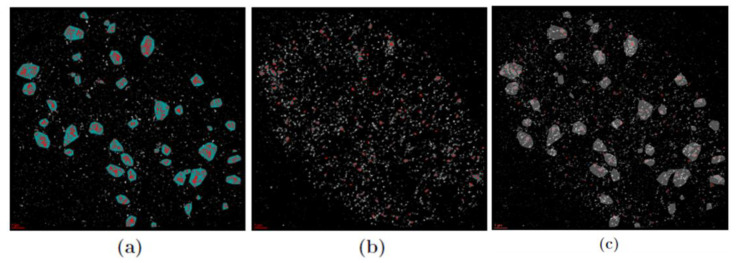
Examples of cluster images of a human skin fibroblast nucleus 60 min after irradiation with 2 Gy. (**a**) γH2AX labeling can be found in clusters highlighted by red points in closed areas. (**b**) MRE11 labeling is dispersed over the cell nucleus and clustered, too. (**c**) The merged image of (**a**) and (**b**) indicating the embedding of MRE11 in γH2AX clusters. Scale bar: 2 µm.

The size of the radiation-induced γH2AX and MRE11 clusters originally determined in [21] were verified by the DBSCAN approach described above (Figure 1). In contrast to γH2AX, which was only found in clusters and foci, MRE11 was not just clustered but additionally dispersed over the cell nuclei (for visual inspection of the foci distribution, see Figure 2). This indicates that MRE11 was available in abundance over the whole repair process observed. Most of the MRE11 clusters were found inside γH2AX clusters (Figure 3), which is in agreement with the literature [21,52]. 

### 3.2. Persistence Homology and Similarities: 1st-Generation Heatmaps

For each of the two cell lines and each time point during repair, the recorded γH2AX and MRE11 clusters were topologically analyzed in terms of persistence homology of zero (components) and one dimension (holes) and the degree of mutual topological similarity was determined. In this way, each single cluster could be compared with another so that, in principle, the 1st-generation heatmaps allowed an overview of the pairwise similarity of all clusters. This might have been useful to roughly investigate the differences of the irradiated cells and the ones without radiation treatment as well as to compare clusters at different times of the repair process. However, to obtain a compact overview of the dynamics of the whole process of cluster formation and relaxation, 1st-generation heatmaps would become large and complex and go beyond the scope of this article. Therefore, 2nd-generation heatmaps (see Section 3.3) had to be considered for this investigation, while 1st-generation ones were limited to the description of a few selected characteristics of damage sites.

**Figure 4:**
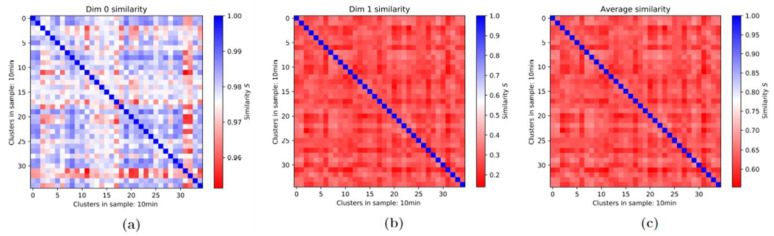
Example of the three 1st-generation heatmaps of γH2AX clusters compared with each other for the control group of the MCF-7 cell line (10 min values). (**a**) The cluster similarities of the components (dim 0), (**b**) those of the holes (dim 1), and (**c**) the average values for each of the pixels from (**a**) and (**b**) are presented. Note: This example is shown because the differences in the similarity of components and holes is large. In the dim 0 heatmap, many clusters have a similarity of above 0.98 and the lowest values are around 0.96. In contrast, in the dim 1 heatmap, the highest value is at about 0.6 and practically all values are in the range of 0.2 to 0.6, excluding the diagonal ones (identity). Therefore, the average is dominated by the dim 1 values. Moreover, the problematic effect of the diagonal can be seen quite effectively as the range of values can only be observed up to a certain degree of accuracy. The differences between the colors for a relatively large spectrum of values are hardly recognizable.

In all cases of comparison of two given samples, three types of heatmaps could be prepared, one for the components (referred to as Dim 0 similarity in the figures), one for the holes (Dim 1), and one of the average values (Average) of dim 0 and dim 1. An example is shown in Figure 4. In this specific case where the clusters detected in the same sample were compared to each other, the heatmap was a square and the diagonal was colored in blue, representing a similarity of one (=identity). It was typical in most of these cases that the components showed a higher degree of topological similarity, while the topological similarity was low for the holes. This reasoned the averaging of the two heatmap data sets and the drawing of conclusions of similarity from the average heatmap. In the case of non-irradiated cells, as shown in Figure 4, the similarity in general was low. The fact that the similarity of the holes is often lower than that of the components can be observed not only in 1st-generation heatmaps but also in 2nd-generation heatmaps and is related to the principle differences of the barcodes of components and holes.

**Figure 5:**
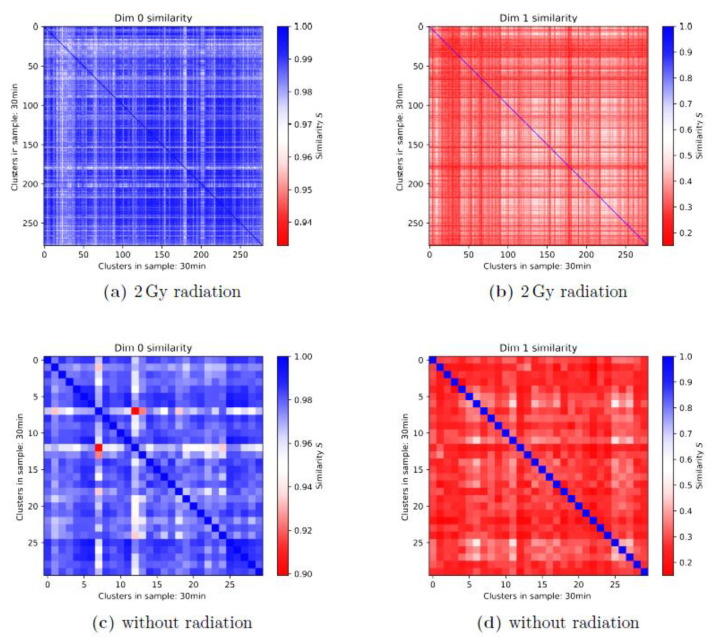
First-generation heatmaps of γH2AX clusters in MCF-7 cells of (**a**,**b**) the irradiated samples (2 Gy) and (**c**,**d**) the non-irradiated control at the time point of 30 min after irradiation. (**a**,**c**) Heatmaps of the components; (**b**,**d**) heatmaps of the holes. Note the different color bars and the significantly different cluster numbers (more than 278 for the irradiated specimen vs. 30 for the non-irradiated specimen).

Using the 1st-generation heatmaps, it was possible to check that there is not only a difference in the cluster size and number between irradiated and non-irradiated cells but also a difference in the topological and geometrical similarity in both components and holes (Figure 5). In this example, the 1st-generation heatmaps compared γH2AX clusters in non-irradiated control MCF-7 cells and the same cells 30 min after exposure to a radiation dose of 2 Gy. The cluster number was significantly higher for the irradiated sample (278 clusters) than for the control sample (29 clusters). Even though the statistical significance within the control group was smaller than within the irradiated group, the difference in similarity was obvious. Except for a few outliers, the bulk of the similarity values for the irradiated sample were above 0.975, while most of the similarity values for the control sample seemed to be in the range of 0.95 to 0.96. In the dim 1 heatmaps, the comparison was easier as the color bars had the same scaling. It is noticeable that the area taken up by white spots, representing a higher similarity than the red spots, was clearly larger for the irradiated sample. This kind of behavior could be observed for all of the different samples.

**Figure 6:**
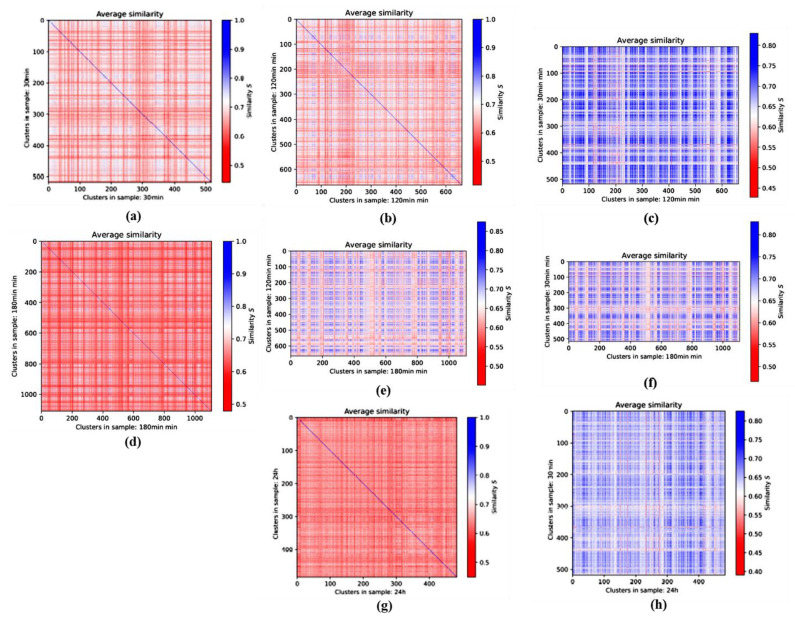
Averaged 1st-generation heatmaps of γH2AX clusters of the skin fibroblast cell line CCD-1059SK. Pairwise comparison of clusters (**a**) 30 min, (**b**) 120 min, (**d**) 180 min, and (**g**) 24 h after irradiation. (**c**) Comparison of 30 min with 120 min clusters, (**e**) 120 min with 180 min clusters, (**f**) 30 min with 180 min clusters, and (**h**) 30 min with 24 h clusters. Note the differences in the color bars of the heatmaps comparing the same time point and comparing different time points.

To obtain a temporal overview of the repair process, one can use different methods in the analysis of the similarity data. First, one can look at two heatmaps of different times post-irradiation and investigate their differences. To exclude effects specific only for particular cancer cells with potentially extensive changes in DSB repair, Figure 6 addresses the effect of repair time on the γH2AX focus topology in normal human skin fibroblasts. Strong differences could be found between the beginning (30 min post-irradiation) and the end (24 h post-irradiation) of the repair process (Figure 6a,g); thereby, the major changes seemed to occur between 120 min (Figure 6b) and 180 min (Figure 6d). In the first 2 h, where most active repair is expected, the overall similarity was highly maintained (Figure 6c), while it was reduced in the next hour (Figure 6e). In this kind of heatmap, the diagonal no longer consists of pixels with value 1 and the diagram is not symmetrical, as two different samples are compared. Hence, the color bar has another resolution. However, those clusters that maintained similarity at 180 min post-irradiation were also highly similar to the clusters at 30 min post-irradiation (Figure 6f). This indicated that if repair continues, it may take place within a maintained topology of the γH2AX cluster. For the 24 h heatmap (Figure 6g), most of the pixels seemed to display a similarity of only 0.6 to 0.7 compared to a great majority of the 30 min heatmap consisting of pixels in the 0.75 to 0.9 area. Considering that the average of the component and hole similarities is displayed in this figure, it is reasonable to conclude that for the 30 min sample with most γH2AX clusters being radiation induced, the overall similarity is much higher than for the 24 h sample in which most radiation-induced γH2AX clusters should have disappeared but other natural reasons, such as proliferation-related damage, should have caused γH2AX cluster formation. In addition, complex, secondarily formed DSB clusters [28] may persist in cells up to 24 h post-irradiation. These clusters are formed by different assemblies of DNA lesions and are therefore irregular. γH2AX foci at this time also diffuse after accomplishing DSB repair, which contributes to their irregularity.

In Figure 6h, the topology of the clusters 30 min post-irradiation and 24 h post-irradiation is compared. As one can see, the values ranged, for the most part, from 0.6 to 0.7, with just a few pixels above 0.75, while also some are of a value of 0.55 and below. This indicates the large dissimilarity of the two samples.

### 3.3. Averaged Similarities along the Repair Period: 2nd-Generation Heatmaps

To obtain more insights into the dynamics of clustering during the repair process of X-ray-induced double-strand breaks, 2nd-generation heatmaps were prepared by calculating the average of every 1st-generation heatmap (where clusters of the same sample were compared) and plotting the result as one pixel of a new heatmap. For this calculation, the diagonal of the 1st-generation heatmaps indicating identity were excluded.

#### 3.3.1. 2nd-Generation Heatmaps of γH2AX

**Figure 7:**
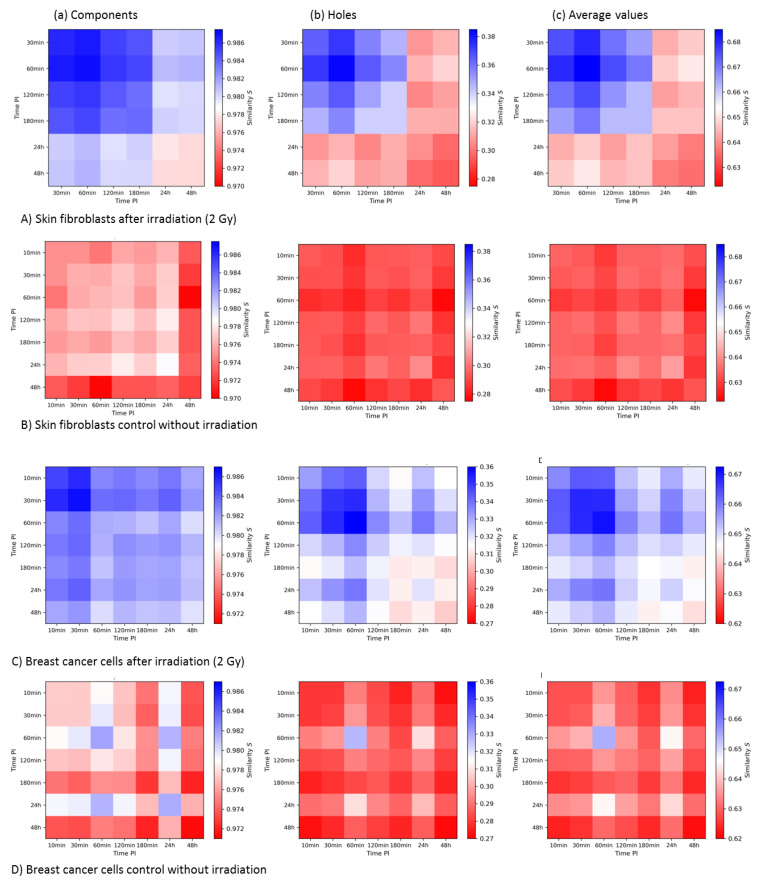
Second-generation heatmaps of γH2AX clusters of components (**a**) and holes (**b**) and average values of both (**c**) for the skin fibroblast cell line CCD-1059SK (**A**,**B**) and the breast cancer cell line MCF-7 (**C**,**D**). Each value of a pixel of these heatmaps represents the mean value of one 1st-generation heatmap. Note the differences in the color bars between (**a**–**c**), while the visual pattern may be similar.

The temporal development of γH2AX cluster similarities in each cell line was investigated. In Figure 7A,B, the results for the irradiated and non-irradiated samples are shown. The color bars of each row (a), (b), and (c) were adjusted to the same range to allow for a better comparison. The 10 min post-irradiation γH2AX values of the irradiated fibroblast samples are missing as this was the only measurement where no clusters were detected. In the irradiated samples, one can clearly see an increased similarity of the detected clusters in the period between 30 min and 180 min post-irradiation for both holes and components. The average values clearly showed that the highest degree of similarity was obtained at 60 min post-irradiation, i.e., the period that corresponds to the maximum numbers of γH2AX foci in most cell types on the micro-scale. The clusters were formed, and the highest repair activity was started with the highest degree of cluster similarity. Although it was shown that at time points of 180 min and later the, number of γH2AX clusters was higher than in the early 2 h (see the former publication about these data [21]), the similarity loss at these later time points indicated that the γH2AX clusters are different from those induced by radiation exposure. Comparing the average values of the irradiated samples at late time points to the non-irradiated samples, it appears to be obvious that in the experiments presented here, only the radiation-induced γH2AX clusters showed an increased similarity, while naturally occurring γH2AX clusters in the control did not. In addition to that, there were no clear temporal dependencies for the non-irradiated samples and the values seemed more randomly distributed. However, it may be considered that the non-irradiated cells were not synchronized, so the different cell cycle state of each cell might have an impact on these data.

With the error of the mean, the statistical errors of the similarity values can be calculated. For each 2nd-generation heatmap, the largest errors were 9.7 × 10^−5^ for the components and 5.6 × 10^−4^ for the holes. Therefore, it could be concluded that the temporal dependencies found in the irradiated samples are of statistical significance, although the statistical error of the holes and thus of the averaged heatmaps was larger in comparison to the components.

Similar differences in the γH2AX focus topology between irradiated and non-irradiated samples and different periods post-irradiation, as reported above for fibroblasts, were observed also for MCF-7 breast cancer cells (Figure 7C,D). Again, the highest degree of similarity was found for γH2AX clusters at 60 min post-irradiation. However, the mutual similarity of individual γH2AX foci in the irradiated cells was generally higher in MCF-7 cells (Figure 7C) than in normal fibroblasts; i.e., it remained higher even at the later post-irradiation time points (Figure 7A). In contrast, when comparing the non-irradiated samples (Figure 7D with Figure 7B), the fluctuations in similarity were much higher for MCF-7 cells than for the fibroblasts. This was also reflected by the statistical error maxima, which were 6.5 × 10^−4^ for the components and 3.1 × 10^-3^ for the holes. Nevertheless, the variations in similarity with time were statistically significant and the similarity values as well as the errors for the holes and averaged values were significantly lower than for the components. 

#### 3.3.2. 2nd-Generation Heatmaps of MRE11

**Figure 8:**
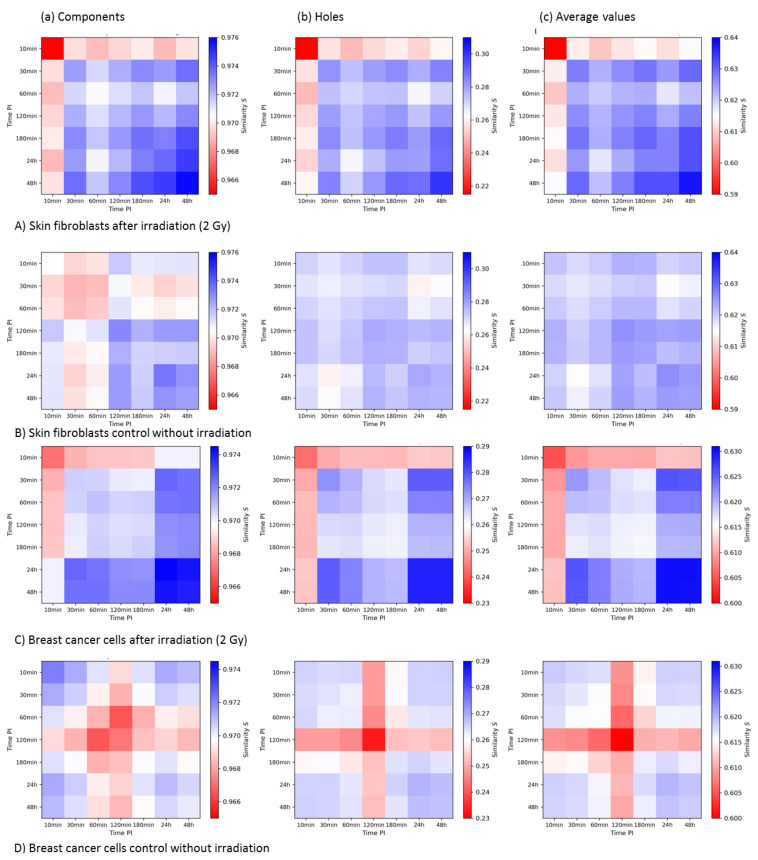
Second-generation heatmaps of MRE11 clusters of components (**a**) and holes (**b**) and average values of both (**c**) for the skin fibroblast cell line CCD-1059SK (**A**,**B**) and the breast cancer cell line MCF-7 (**C**,**D**). Each value of a pixel of these heatmaps represents the mean value of one 1st-generation heatmap. Note the differences in the color bars between (**a**_–_**c**), while the visual pattern may be similar.

In Figure 8, the 2nd-generation heatmaps of MRE11 in the irradiated (Figure 8A) and non-irradiated (Figure 8B) skin fibroblast samples are shown. The color bars of the components and holes and average values were adjusted to each other to compare the irradiated and control samples more accurately.

The 10 min sample showed the lowest similarity values in all cases. For the samples in the period of 30 min to 180 min post-irradiation, the similarity seemed to increase slightly. This may be correlated to the expected repair dynamics with an increase in similar MRE11 clusters during the later times of repair. However, the largest values occurred at 48 h post-irradiation. 

In contrast, although the control samples showed some similarities, too, there was, in general, no clear tendency or obvious time course. When comparing irradiated and non-irradiated samples, one could notice an overall higher similarity for the components in clusters for the irradiated samples. The maximum statistical errors, given by the error of the mean, were 3.2 × 10^−2^ for the components and 1.5 × 10^−4^ for the holes. 

The results for the MRE11 cluster similarities in the MCF-7 breast cancer cell line are shown in Figure 8C,D. From the average values (c), it could be concluded that the highest degree of similarity can be found at 24 h and 48 h. However, these clusters were similar to clusters at 30 min. The maximum statistical errors were given by 2.6 × 10^−4^ for the components and 1.5 × 10^−3^ for the holes. Considering these statistical errors, the differences of the values of the heatmaps are of statistical significance.

#### 3.3.3. Summary and Comparison of 2nd-Generation Heatmaps of γH2AX and MRE11

The post-irradiation time dependency of topological cluster similarity differed strongly for the two types of proteins. γH2AX clusters showed a clear increase in similarities at earlier time points, when the process of photon-induced double-strand break repair is expected to be ongoing. In contrast, MRE11 cluster similarity increased at later post-irradiation times and peaked at time points when persistent DSBs are expected. Both proteins showed a slightly different behavior for the different cell lines. For γH2AX, the increase in similarity at earlier time points post-irradiation was limited to a shorter time interval in the fibroblast cell line compared to the breast cancer cell line. The similarity values of MRE11 clusters showed a more homogeneous distribution for the 30 min to 180 min time period in the fibroblasts. In general, the similarity values were significantly higher for γH2AX clusters than for MRE11 clusters. The MRE11 cluster size and the number of detected signals per cluster were significantly smaller compared to γH2AX. This has to be taken into consideration when evaluating the results.

## 4. Discussion

The aim of this article was to obtain first insights into the potential relevance of spatial organization of chromatin during DNA double-strand break repair processes after irradiation with X-rays in breast cancer cells and normal skin fibroblasts. The formation of γH2AX was investigated with regard to the internal spatial arrangement/topology of the formed foci; in the same way, MRE11 as one of the initiators of the homologous recombination repair pathway was analyzed. Besides the qualitative observations of cluster number and size, the topological structure of γH2AX and MRE11 clusters was evaluated. 

To verify the cluster formation and to obtain suitable data sets for topological analyses, the data acquired in [21] were re-evaluated by the cluster detection algorithm DBSCAN using interactively adjusted parameters for cluster definition. This was achieved by computing cluster images for various parameters and judging qualitatively whether reasonable clusters as well as no background signals were detected as cluster points. By using a closing function, the results could precisely verify the original clustering [21]. Qualitative analysis of sizes and numbers of clusters in different samples led to reasonable conclusions. The clusters of γH2AX and MRE11 differed strongly as significantly more clusters were detected for MRE11 with a significantly smaller size. This is compatible to other analyses of other radiation quality [47] and the molecular processes known for MRE11 [47]. In general, clusters in different cell lines of the same protein did not differ in size, but the number of detected clusters was higher in fibroblasts than in breast cancer cells. 

For topological analysis using persistent homology, it is important to note that for heatmaps, a difference in topological similarity can also be caused by changes in cluster size and does not directly imply a different sub-structure. However, in the case of cluster formation discussed here, the size difference between radiation-caused clusters and the ones around naturally occurring DSBs is expected to be small [21].

For γH2AX clusters in fibroblasts, a significant increase in topological similarity was found 30 min to 180 min post-irradiation. This indicates that around photon-radiation-induced DSBs, γH2AX clusters appear to be formed in a characteristic manner, which was not observed in the 24 h to 48 h samples and non-irradiated samples, where only naturally occurring DSBs should be present. 

It should be mentioned that different phases of the cell cycle could influence the number and spatial arrangement of the investigated clusters [55]. During DNA replication also, DSBs could occur. As it is not known at which state of the cell cycle the investigated cells individually were detected, this might lead to some unexpected observations. For example, the 48 h sample of the non-irradiated fibroblasts showed a significantly lower similarity than all other samples, which might be due to the mentioned cell cycle effect. 

In the MCF-7 cell line, comparable observations were made. The 10 min and 30 min samples had a significant increase in γH2AX cluster similarity compared to the 120 min to 48 h samples. Again, this suggests a characteristic formation of γH2AX clusters around photon-induced DSBs. In contrast, the results of fibroblasts with an abrupt decrease between the 30 min and 60 min samples indicated a considerably shorter repair time. In principle, γH2AX clusters in healthy fibroblasts and cancer cells differ in the time range of increased similarity after irradiation, which may be compatible with a shortened repair time of photon-induced DSBs in fibroblasts. In both normal and cancer cell lines, the similarity is significantly increased for short post-irradiation times and it drops down to the level of non-irradiated cells afterward. 

As part of the MRN complex, MRE11 is a critical protein involved in the ignition of repair of DNA double-strand breaks. Furthermore, it promotes the HR repair pathway, since MRE11-deficient cells have a decreased efficiency of HR compared to cells with normal levels of MRE11 [56]. However, the investigation of the MRE11 topology under the aspect of the repair pathway decision toward HR can only be the first step toward understanding the relevance of the chromatin/repair focus topology for the repair pathway decision. Further investigations of the topology of Rad51 clustering [57] in comparison to the MRE11 cluster topology may be the next step and give deeper insights into this subject. Such investigations will therefore be envisaged in future research.

For fibroblasts, the similarity of MRE11 clusters increased at later post-irradiation times. The similarity values for short post-irradiation times seem to be randomly distributed; only for 180 min and longer post-irradiation, a clear, gradual increase in similarity could be observed. In comparison to non-irradiated cells, the similarity values of MRE11 were significantly higher. As only naturally occurring DSBs are expected in control samples, it seems that radiation-induced DSBs cause the formation of MRE11 clusters in a characteristic way. In [21], the analysis of MRE11 clusters in fibroblasts suggested a conformational opening for homologous recombination within a cluster, which led to further changes in chromatin arrangement of the nucleus. This could explain the strong fluctuations in similarity for short post-irradiation times. In addition, the cluster number continually increases until long post-irradiation times and peaks at 48 h. Together with the observation that the similarity of clusters also peaks at this time, this result indicates long time effects of photon radiation, leading to characteristic and persistent MRE11 clusters. The nature of these clusters may be different from DSB repair clusters and should be further investigated. Another factor that could have influenced the similarity is the cell cycle state of the individual cell.

It might also be considered that at the early periods of post-irradiation time, H2AX phosphorylation only occurs at organized chromatin, while MRE11 is a mixture of free MRE11 protein aggregates and already bound MRE11 to DSB sites. With increasing post-irradiation time, there exists less free MRE11 and more bound protein. Nevertheless, as a component of the MRN complex, MRE11 binds to DSB sites fast after irradiation. As MRE11 binds to broken DNA ends and forms only smaller foci at the early post-irradiation time points, the similarity may be small. With increasing post-irradiation time, the free MRE11 reduces, while the size of MRE11 foci may increase. However, this explanation may only be part of the explanation of the similarity at late time points. Moreover, the dynamic actions and interactions of MRE11, BRCA1, and CtIP as multi-meres and individual molecules have to be considered, as recently shown in combination with the recruitment and spatial exclusion of 53BP1 [58]; thereby the role of helicases and nucleases as well as inhibitory mechanisms of MRE11 exo-/endonuclease inhibition may round up the image of multi-factor interaction mechanisms [58].

MRE11 clusters in the MCF-7 cell line showed a similar behavior as in the fibroblast cell line. The lowest values for cluster similarities of components were found in the 10 min sample, with slightly increased but homogeneous similarities in the 30 min to 180 min samples. The highest values could be found at 24 h and 48 h. A clear response of the cells to radiation was observed. In [21], the behavior of MRE11 clusters at short post-irradiation times was interpreted as a conformational change of the DNA due to the high numbers of clusters detected. This could explain the observations regarding the similarity in the 10 min to 180 min samples. As in the fibroblasts, the significant peak at later post-irradiation times suggested long time effects of the radiation. This seems to cause the formation of persistent and highly similar clusters.

In conclusion, MRE11 clusters show similar behavior in fibroblasts and breast cancer cells. Especially at 24 h and 48 h, the development seems independent of the cell line and is noticeable in both components and holes. This suggests the existence of persistent clusters with increased similarity in these times caused by the effects of photon radiation.

The results presented here indicate the potential of the nano-architecture of chromatin for DNA DSB repair processes and show that the presentation of damage sites is non-randomly organized. Therefore, one may hypothesize that the size and shape as well as the tempo-spatial organization of the γH2AX and early repair protein clusters have an impact on the accessibility to the damaged site for additional repair proteins. The spatial arrangements of the downstream repair proteins seem to be a consequence of this spatially selected accessibility. Variation in radiation dosage, radiation type, post-irradiation time selection, and examined cell lines might thus be useful to obtain a deeper insight into the processes involved. In addition, it would be interesting to further investigate the role of the cell cycle in the behavior of the repair proteins involved.

## 5. Conclusions

DNA double-strand breaks are the most severe type of DNA damage, which justifies research into the mechanisms involved in the repair of these breaks [1,2,5,6,17,58]. The aim of this article was to obtain a deeper insight into the structural arrangements of γH2AX and MRE11 clusters after irradiation of cells with X-rays. A novel cluster detection algorithm was applied on data gathered by SMLM. The detected clusters were compared in terms of their topological similarity in order to investigate the functional potential of spatial chromatin arrangements during repair. It was found that in breast cancer cells as well as in human skin fibroblasts, the DNA damage response causes an increase in cluster similarity. In the case of γH2AX clusters, a clear time interval of increased similarity in the early post-irradiation period was observed. For MRE11 clusters, the increased similarity peaked at long periods after irradiation. For both proteins, the differences from naturally occurring clusters in the non-irradiated samples were clearly noticeable. This indicates that the spatial arrangements of repair proteins in both cell lines may be depending on the way the damage is caused and repaired. The different cell lines show different reaction times to the induced damage. In irradiated samples, MRE11, an indicator of HRR, was detected in both cell lines, with significant topological differences from the non-irradiated control cells. This might suggest that for a dose of 2 Gy, HRR could be a promoted repair process for the photon induced DSBs, as already proposed in [59]. The use of SMLM in analysis of DNA damage repair has been proven to be a powerful tool to investigate the proteins involved and the repair pathway chosen.

## Data Availability

Data are part of the KIP SMLM data archive and can be obtained upon request to the corresponding author.
